# Gut Microbiota Modulation in Osteoporosis: Probiotics, Prebiotics, and Natural Compounds

**DOI:** 10.3390/metabo15050301

**Published:** 2025-04-30

**Authors:** Xufeng Chu, Hailin Xing, Minghao Chao, Panpan Xie, Lili Jiang

**Affiliations:** 1 Department of Orthopedic Surgery, The Fifth Affiliated Hospital of Wenzhou Medical University, Lishui Municipal Central Hospital, 289 Kuocang Road, Lishui 323000, China; doctorchuxf@163.com (X.C.); lishuixhl@163.com (H.X.); 17772293609@163.com (M.C.); 2 Department of Laboratory Medicine, The Fifth Affiliated Hospital of Wenzhou Medical University, Lishui Municipal Central Hospital, 289 Kuocang Road, Lishui 323000, China

**Keywords:** gut microbiota, modulation, osteoporosis, natural compounds

## Abstract

Osteoporosis is a multifactorial bone metabolic disorder characterized by the deterioration of bone mass and microarchitecture, leading to increased fragility and fracture risk. Recent advances have revealed the critical role of the gut microbiota in the pathogenesis of osteoporosis, primarily mediated by metabolite-driven and immune-mediated interactions along the gut–bone axis. Dysbiosis, or microbial imbalance, can influence bone health by modulating host metabolism, immune function, and endocrine responses. While growing evidence suggests that gut microbiota modulation holds therapeutic potential for osteoporosis, the underlying mechanisms remain poorly understood. This review examines the latest findings on the role of prebiotics, probiotics, and natural bioactive substances in modulating the gut microbiota to improve bone health. We discuss how these interventions may restore microbial balance, enhance gut barrier function, and reduce systemic inflammation, thereby influencing bone metabolism. A deeper understanding of the gut–bone axis will pave the way for more targeted, effective, and personalized therapeutic strategies for osteoporosis prevention and treatment.

## 1. Osteoporosis and Gut Microbiota

### 1.1. Osteoporosis

Osteoporosis is a systemic skeletal disorder characterized by reduced bone mass and the deterioration of bone microarchitecture, which increases bone fragility and susceptibility to fractures. The pathogenesis primarily involves an imbalance in bone metabolism, where bone resorption exceeds bone formation, leading to progressive bone loss. Osteoporosis is most commonly seen in the elderly, particularly postmenopausal women and older men, as bone density decreases with age [1]. According to the World Health Organization (WHO), approximately 41.5 million people worldwide are affected by osteoporosis, making it a major global health issue [2].

Primary osteoporosis arises from age-related bone loss or estrogen deficiency (e.g., postmenopausal osteoporosis), while secondary osteoporosis results from medical conditions (e.g., hyperparathyroidism) or medications (e.g., glucocorticoids). Risk factors include genetic predisposition, age, gender, hormonal changes, poor nutrition (e.g., insufficient calcium and vitamin D), lack of physical activity, smoking, and excessive alcohol consumption [3]. Additionally, medical conditions such as hyperparathyroidism, rheumatoid arthritis, and long-term use of glucocorticoids also increase osteoporosis risk [4,5]. Osteoporosis is often asymptomatic, with many individuals unaware of their condition until they experience a fracture. Given its gradual and painless progression, osteoporosis is sometimes referred to as the “silent killer” or “iceberg disease” [6]. Globally, osteoporosis is responsible for 8.9 million fractures annually, with common fracture sites including the spine, hip, and forearm, and an increasing frequency of fragility fractures in the pelvis [7].

### 1.2. Gut Microbiota

The gut microbiota is a complex microbial ecosystem comprising bacteria, archaea, viruses, and fungi that reside in the human digestive tract. In adults, approximately 100 trillion microorganisms make up the gut microbiota, whose collective genetic material, the microbiome, vastly outnumbers the human genome. This microbiome is often referred to as the “second genome” [8]. In recent years, attention has focused on the gut microbiota’s role in maintaining host health and regulating disease processes, including metabolic, immune, nervous, and endocrine functions.

Metabolically, the gut microbiota breaks down dietary fibers and polysaccharides that the host cannot digest, producing short-chain fatty acids (SCFAs) like acetate, propionate, and butyrate. These metabolites serve as important energy sources for intestinal epithelial cells and modulate immune responses and energy metabolism [9]. The microbiota also synthesizes essential amino acids, vitamins (such as vitamins K and B), and regulates bile acid and lipid metabolism, playing a critical role in maintaining metabolic balance [10,11]. Additionally, the gut microbiota is crucial for the development and function of the immune system, interacting with intestinal immune cells to modulate both innate and adaptive immune responses [12,13].

Homeostasis of the gut microbiota is vital for health. Dysbiosis, or an imbalance in microbial populations, is linked to various diseases, including obesity, diabetes, inflammatory bowel disease, allergic disorders, depression, neurodegenerative diseases, and certain cancers [14]. Dysbiosis is typically marked by a reduction in beneficial microbes, an overgrowth of pathogenic species, and decreased microbial diversity. Factors such as poor diet, overuse of antibiotics, infections, stress, and lack of physical activity can trigger dysbiosis [15].

In summary, the gut microbiota, often referred to as an “invisible organ” [16], plays a crucial role in both physiological and pathological processes. Further exploration of the relationship between the microbiota and host health could lead to novel therapeutic strategies and personalized health management approaches.

### 1.3. Gut–Bone Axis

Recent studies have emphasized the pivotal role of the gut microbiota in regulating bone metabolism through the “gut–bone axis”. Dysbiosis significantly influences bone health by modulating the host’s metabolic, immune, and endocrine systems, contributing to the development and progression of osteoporosis and bone loss [17,18]. The growing recognition of the gut microbiota as a potential regulator of bone health highlights its complex and multifaceted interactions with bone metabolism.

The gut microbiota can directly or indirectly affect bone mass through various mechanisms, including the modulation of host metabolism, calcium absorption, hormone levels, immune function, and the central nervous system [17,19]. However, despite progress in understanding the gut–bone axis, the underlying mechanisms remain poorly defined. The bidirectional interactions between bone and the gut microbiota, and the specific regulatory pathways involved, require further investigation. This gap in knowledge hinders our understanding of osteoporosis pathogenesis and limits the development of microbiota-based interventions.

Nevertheless, the gut microbiota presents significant potential as a therapeutic target for osteoporosis and related bone diseases. Microbiota-based approaches, such as probiotics, prebiotics, and natural bioactive compounds, could offer effective strategies for preventing and treating osteoporosis. These interventions aim to restore a healthy microbial balance early, suppress bone disease, and explore new avenues for osteoporosis prevention and treatment.

### 1.4. Modulation of Gut Microbiota

Gut microbiota modulation can be achieved through various interventions aimed at restoring microbial balance and optimizing host health. Key strategies include dietary changes, probiotics, prebiotics, antibiotics, FMT, and bioactive compounds [20]. These approaches influence microbiota composition and function, thereby impacting host metabolism, immune responses, and overall health.

Studies have identified significant differences in the gut microbiota of osteoporosis patients compared to healthy controls [21]. Targeting the gut microbiota in osteoporosis has thus emerged as an important area of research. Investigating gut microbiota–bone interactions can help uncover the pathophysiological mechanisms of osteoporosis and provide a scientific foundation for developing new therapeutic strategies.

Probiotics and prebiotics, as key modulators of the gut microbiota, have been shown to significantly influence bone metabolism. Probiotics enhance bone health by increasing beneficial gut bacteria, restoring microbial balance, and modulating immune responses to reduce inflammation in bone metabolism. For example, *Lactobacillus rhamnosus GG* has been shown to regulate the dysbiosis in the gut of ovariectomized mice. This probiotic promotes the secretion of anti-inflammatory cytokines, such as TGF-β and IL-10, while suppressing pro-inflammatory cytokines like TNF-α and IL-17. These effects help mitigate estrogen deficiency-induced inflammation, reduce osteoclastic activity, and improve osteoporosis [22].

Prebiotics, which serve as a “fuel” for probiotics, indirectly support bone health by promoting the growth and activity of beneficial gut bacteria. The fermentation of prebiotics in the gut produces short-chain fatty acids (SCFAs), such as butyrate, that activate gut receptors to inhibit bone resorption and stimulate bone formation [23]. Additionally, prebiotics improve intestinal barrier function, reduce gut permeability, and limit the passage of harmful bacteria and their toxins, further protecting bone health [24].

In addition, plant-derived natural compounds, particularly polyphenols and flavonoids, are increasingly recognized for their ability to regulate the gut microbiota and improve bone health. These compounds positively influence bone metabolism through various mechanisms. Oxidative stress is a key factor in osteoporosis, and polyphenolic compounds have strong antioxidant properties that help alleviate oxidative damage. Moreover, grape seed extract has been shown to modulate gut microbiota composition, increase beneficial bacterial populations, and alter their metabolic profiles, thereby protecting bone mineral density and slowing osteoporosis progression [25].

In summary, the regulation of gut microbiota plays a pivotal role in bone metabolism and may significantly influence the onset and progression of osteoporosis. While current evidence supports the potential of probiotics, prebiotics, and plant-derived compounds in bone health, further investigation is needed to understand the underlying mechanisms and optimize these strategies for clinical applications.

### 1.5. Potential Mechanisms of Gut Microbiota in Bone Metabolism

Although substantial evidence supports the link between gut microbiota and bone metabolism, the exact molecular mechanisms remain insufficiently understood. Key questions persist regarding the roles of microbial metabolites, signaling pathways, and immune modulators in maintaining bone health.

**Figure 1:**
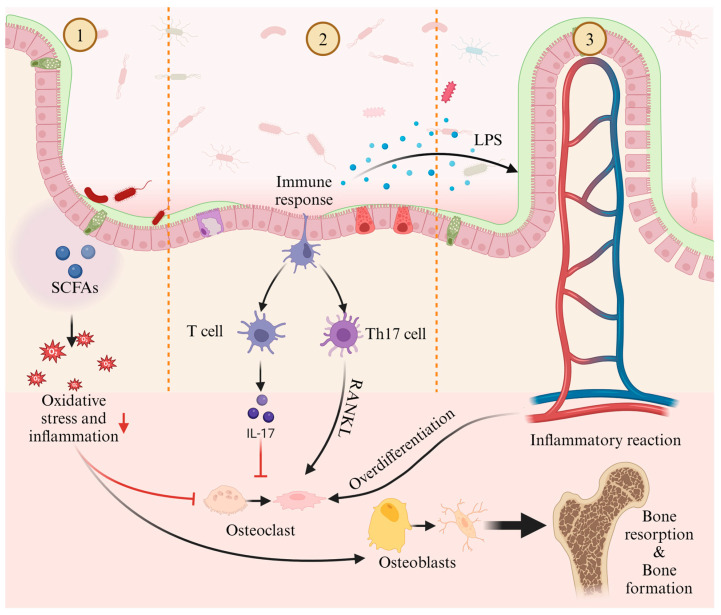
Potential mechanisms of gut microbiota in regulating osteoporosis: ① The metabolites of gut microbiota, short-chain fatty acids, modulate osteoblasts and osteoclasts through oxidative stress and inflammatory responses, affecting bone resorption and bone formation; ② Gut microbiota influences bone resorption by regulating the balance between T cell and Th17 cell; ③ Inflammatory factors, such as LPS, which are metabolites of gut microbiota, excessively activate osteoclasts through inflammatory responses, impacting bone resorption and bone formation.

One significant mechanism by which gut microbiota may influence bone homeostasis is through the diffusion of intestinal metabolites into systemic circulation (Figure 1, Part ①). Among these metabolites, short-chain fatty acids (SCFAs) such as butyrate, propionate, acetate, and valerate have been central to gut–bone axis research [26]. For example, valerate supplementation in ovariectomized mice has been shown to reduce bone resorption and improve bone microstructure [27]. Valerate exerts its effects primarily by inhibiting RELA protein production and enhancing IL-10 mRNA expression, which suppresses osteoclast maturation while promoting osteoblast differentiation. Similarly, sodium butyrate has been shown to alleviate oxidative stress and inflammation in rats, promoting osteoblast differentiation and inhibiting osteoclast differentiation [28]. Additionally, sodium butyrate enhances SIRT1 expression, a NAD^+^-dependent deacetylase that mitigates oxidative stress and promotes osteoblast differentiation while concurrently inhibiting histone deacetylases (HDACs) [17,28]. This dual action restores bone metabolism by balancing acetylation-dependent signaling, ultimately improving bone strength and density in osteoporotic models [17]. Taken together, these data suggest that SCFAs are effective regulators of bone cell metabolism and play a significant role in maintaining bone homeostasis [29].

Furthermore, gut microbiota interact with immune cells to regulate both innate and adaptive immune responses [30] (Figure 1, Part ②). CD4+ T helper (Th) cells play a pivotal role in immune function, modulating other immune cells, such as B lymphocytes, through surface receptors and cytokines. In the gut, Th cell expansion facilitates their migration to the bone marrow, where they increase the recruitment of inflammatory monocytes as osteoclast precursors [31]. Imbalances between Th17 and regulatory T (Treg) cells have been linked to bone health, as Th17 cells promote osteoclast differentiation and bone resorption via the RANKL pathway, while Treg cells suppress osteoclast differentiation [32].

Finally, increased intestinal permeability allows inflammatory mediators, such as lipopolysaccharides (LPS), to enter systemic circulation, triggering inflammation and excessive osteoclast activation (Figure 1, Part ③). LPS has been implicated in postmenopausal osteoporosis, where it promotes femoral bone loss and enhances osteoclast survival while inhibiting osteogenic differentiation of osteoclast precursors [33,34].

**Figure 2:**
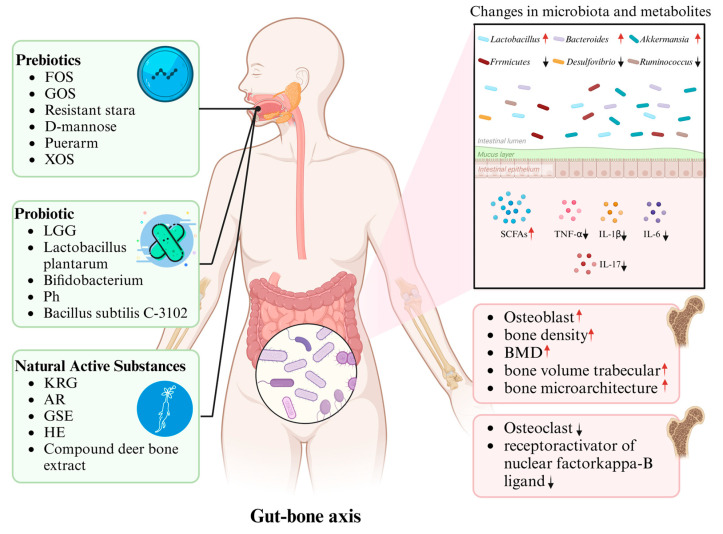
The pivotal role of gut microbiota in mediating the effects of prebiotics, probiotics, and natural substances on osteoporosis regulation: Upon ingestion, these bioactive agents modulate the composition and relative abundance of gut microbiota, thereby inducing alterations in metabolite profiles, which subsequently drive structural bone remodeling and contribute to the amelioration of osteoporosis.

Understanding these mechanisms is critical for developing microbiota-targeted therapies. Therefore, the following sections explore research progress on the role of prebiotics, probiotics, and natural bioactive compounds in modulating the gut microbiota to improve osteoporosis, laying the foundation for further investigations into the specific mechanisms of these interventions, their impacts on the gut microbiota, and the development of more precise and personalized intervention strategies (Figure 2).

### 1.6. NAFLD/MAFLD and Its Emerging Role in Bone Metabolism

Non-alcoholic fatty liver disease (NAFLD) is a multisystemic condition and has become the leading cause of chronic liver disease worldwide, affecting approximately 25% of the global population and posing a significant burden on healthcare systems [35]. To better reflect its metabolic underpinnings, an international panel of experts has proposed renaming NAFLD as metabolic dysfunction-associated fatty liver disease (MAFLD) [36].

NAFLD/MAFLD is increasingly recognized not only as a liver-centric disease but also as a condition with systemic implications. A growing body of epidemiological evidence has established a link between NAFLD/MAFLD and osteoporosis. Patients with NAFLD exhibit a significantly higher risk of developing osteoporosis [37]. In a U.S. population-based study involving individuals aged 20–59 across various races and sexes, NAFLD was independently associated with reduced bone mineral density (BMD) [38]. Similarly, Chinese cohort studies have shown a consistent inverse relationship between NAFLD and BMD, regardless of sex [39,40]. These findings suggest that NAFLD contributes to osteoporosis not only through impaired hepatic function but also via systemic metabolic alterations. Proposed mechanisms include the dysregulation of bone turnover markers, vitamin D deficiency, chronic hepatic inflammation, liver fibrosis, a disrupted lipid metabolism, and gut microbiota dysbiosis [41].

Dysbiosis of the gut microbiota is frequently observed in patients with NAFLD. Altered microbial composition compromises intestinal barrier integrity, allowing bacterial metabolites such as lipopolysaccharide (LPS), short-chain fatty acids (SCFAs), and ethanol to translocate via the portal circulation to the liver, thereby exacerbating hepatic metabolic dysfunction through the gut–liver axis [42]. In parallel, chronic low-grade inflammation and metabolic disturbances arising from gut dysbiosis can affect bone metabolism via the gut–bone axis, as demonstrated in models of osteoporosis [43].

These observations highlight a complex bidirectional interaction wherein liver health—particularly in the context of NAFLD—modulates gut microbiota composition, which, in turn, influences bone homeostasis. The gut–liver–bone axis has thus emerged as a potential physiological network of critical importance in bone metabolism regulation. Elucidating the mechanistic pathways underlying this axis may offer novel strategies for the prevention and treatment of osteoporosis in individuals with metabolic liver disease [44].

### 1.7. Sex Differences in Gut Microbiota and Osteoporosis Pathogenesis

Significant sex-related differences exist in gut microbiota composition, bone metabolism, and the pathogenesis of osteoporosis. In females, hormonal fluctuations related to the menstrual cycle and estrogen levels induce unique gut microbiota patterns, distinct from those observed in males [45]. Regarding bone metabolism, females generally exhibit a higher rate of bone formation and a lower rate of bone resorption, primarily due to the influence of estrogen [46]. However, with aging and the associated decline in estrogen levels, females become more susceptible to osteoporosis. In contrast, males experience relatively stable estrogen levels, with bone metabolism being predominantly influenced by androgens and other metabolic factors [47]. Although osteoporosis occurs less frequently in males, their bone mineral density significantly declines with aging, especially when testosterone levels decrease [48]. Estrogen not only affects gut microbiota composition but also modulates bone metabolism via specific immune pathways within the gut–bone axis. Therefore, the sex-dependent regulation of bone metabolism is likely linked to differences in gut microbiota composition [49].

Reproductive states such as pregnancy, lactation, and menopause exert profound effects on both gut microbiota and bone metabolism in women. During pregnancy and lactation, significant hormonal changes influence both bone metabolism and gut microbiota composition. Studies have shown that during pregnancy, hormonal levels of estrogen and progesterone rise significantly, leading to changes in gut microbiota diversity. These changes are closely associated with maternal bone metabolism [50]. Moreover, during pregnancy, increased calcium release from bones supports fetal skeletal development, yet this process also elevates the risk of osteoporosis [51]. Postmenopausal women experience a sharp decline in estrogen levels, leading to significant reductions in bone mineral density and an increased risk of osteoporosis. This decline in estrogen is closely linked to heightened inflammation and increased bone resorption [52]. Importantly, postmenopausal women often exhibit gut dysbiosis, and the decrease in estrogen levels not only directly affects bone metabolism but also indirectly influences osteoporosis by modulating gut microbiota and the immune system [53].

### 1.8. Literature Search Strategy

A systematic search was conducted across three major databases (PubMed, Web of Science, and Scopus) for studies published between January 2010 and March 2024, using the following Boolean search terms: (“gut microbiota” OR “gut microbiome” OR “intestinal flora”) AND (“osteoporosis” OR “bone loss” OR “bone mineral density”) AND (“prebiotics” OR “probiotics” OR “synbiotics” OR “natural compounds” OR “bioactive substances”). Inclusion Criteria: (1) Study types: peer-reviewed randomized controlled trials (RCTs), preclinical studies (animal models or in vitro mechanistic investigations), or observational studies with mechanistic analyses; (2) Outcomes: Focus on bone health parameters, including but not limited to bone mineral density, bone microstructure, or biochemical markers. Exclusion Criteria: (1) non-English publications; (2) studies without control groups; (3) review articles, commentaries, or meta-analyses lacking original experimental data.

## 2. Prebiotics and Osteoporosis

In 1995, Gibson and Roberfroid introduced the concept of prebiotics—non-digestible food ingredients that selectively stimulate the growth and/or activity of beneficial gut bacteria, thereby improving host health [54]. Clinical studies have shown that prebiotic supplements, including fructooligosaccharides (FOS) and galactooligosaccharides (GOS), can reduce body fat and alleviate obesity-related diseases [55,56]. Moreover, prebiotics have been reported to enhance calcium retention in postmenopausal women and increase bone mass in ovariectomized rats [57,58]. This has led to growing interest in the role of prebiotics in preventing bone loss and osteoporosis, as well as in understanding the underlying mechanisms (Table 1, Figure 2).

The protective effects of FOS on osteoporosis have been validated in several animal studies. Tanabe et al. fed high-fat diet (HFD) mice 5% FOS and observed significant improvements in osteoporosis and changes in gut microbiota composition [24]. Compared to the control group, FOS treatment alleviated HFD-induced bone loss, reversed the imbalance in differentiation between osteoblasts, adipocytes, and osteoclasts, and improved gut barrier function by reducing tight junction protein downregulation and inflammatory factor increases. These effects were associated with a correction of gut dysbiosis, including an increase in the *Firmicutes*/*Bacteroidetes* ratio and a decrease in microbial diversity. Similar findings were observed in accelerated aging male mice, where FOS supplementation led to higher bone calcium content and an increased abundance of *Lactobacillus* and *Bacteroides* compared to the control group [59]. In female SD rats, short-chain FOS supplementation increased bone metabolic response and peak bone mass compared to ovariectomized rats [23].

GOS, a naturally occurring prebiotic found in human milk, has also shown potential in improving mineral balance and bone characteristics. Weaver et al. investigated the effects of varying concentrations of GOS on bone mineral content and properties in rats [60]. GOS supplementation resulted in significantly higher mineral absorption and retention, including calcium and magnesium, compared to controls. Additionally, femur and tibia breaking strength, as well as bone mineral density (BMD) at both the distal and proximal femur ends, were significantly increased. The relative abundance of *Bifidobacteria* also increased, while microbial diversity decreased. Human studies by Whisner et al. on GOS supplementation in girls aged 10–13 years confirmed these findings, with GOS significantly improving calcium absorption and increasing *Bifidobacterium* abundance in fecal samples [66]. These results suggest that GOS-enhanced calcium absorption is mediated through *Bifidobacteria*.

In addition to FOS and GOS, other prebiotics such as resistant starch, D-mannose, puerarin, and xylooligosaccharides (XOS) have also been studied for their impact on bone resorption and loss. Tousen et al. fed female mice with resistant starch (cornstarch) to assess its effects on bone loss [61]. Compared to the control group, mice on a 12% resistant starch diet showed reduced bone loss following ovariectomy, with a downregulation of RANKL and interleukin-7 receptor gene expression in bone marrow. These effects were likely linked to increased IL-10 expression in colon tissues and an enhanced relative abundance of *Bifidobacteria*. In a follow-up study, resistant starch supplementation significantly reduced bone resorption and increased *Bifidobacterium* levels in fecal samples [62]. Similarly, Liu et al. found that D-mannose supplementation in mice significantly improved cortical bone volume and trabecular bone microstructure while downregulating osteoclastogenesis-related factors [63]. The gut microbiota of D-mannose-fed mice showed increased abundances of *Firmicutes*, *Erysipelotrichales*, *Verrucomicrobia*, and *Akkermansiaceae* compared to control and ovariectomized groups.

Studies on puerarin, a natural compound, have demonstrated its ability to increase bone density in rats, improve gut dysbiosis, and enrich gut microbial metabolism related to amino acids, lipopolysaccharide (LPS) biosynthesis, and butyrate production, while also increasing short-chain fatty acid concentrations [64]. XOS supplementation has similarly been shown to increase bone density, breaking strength, bone crystallinity, and calcium transport protein expression in mice [65]. These findings indicate that prebiotics, as dietary components, can modulate gut microbiota structure and function, influencing bone resorption and improving bone characteristics.

Through the analysis and summary of different experimental models, it was found that in the majority of ovariectomized mouse models, supplementation with prebiotics primarily increased the relative abundance of *Bifidobacteria* in the gut, as well as the concentration of short-chain fatty acids, amino acid metabolism, biosynthesis of lipopolysaccharides (LPS), and pyrimidine metabolism. This also led to a reduction in systemic inflammation and the regulation of bone metabolism. In high-fat diet-induced obese mouse models, prebiotic supplementation mainly increased the *Firmicutes–Bacteroidetes* ratio in the gut, improved intestinal barrier function, and reversed the imbalance in the differentiation of osteoblasts, adipocytes, and osteoclasts, thereby modulating bone metabolism. In growth-stage or preclinical models during the growth phase, prebiotic supplementation mainly increased the relative abundance of *Bifidobacteria* in the gut and enhanced calcium absorption in the bones. In accelerated aging mouse models, prebiotic supplementation led to an increased relative abundance of *Lactobacillus*, *Bacteroides*, and *Clostridium* spp. in the gut.

In summary, prebiotics such as FOS, GOS, resistant starch, D-mannose, and XOS have been shown to modulate gut microbiota composition and improve bone health through several interconnected mechanisms. These prebiotics primarily promote the growth of beneficial bacteria, including *Bifidobacteria* and *Lactobacilli*, which are associated with enhanced gut barrier function and reduced systemic inflammation. The microbiota alterations induced by prebiotic treatments lead to increased production of SCFAs, such as butyrate, which play a vital role in maintaining intestinal integrity and reducing inflammation. Additionally, some prebiotics improve calcium and mineral absorption in the gut, potentially by enhancing the microbiota-mediated breakdown of dietary fibers and boosting mineral bioavailability. In animal studies, prebiotics like FOS and GOS have demonstrated the ability to modulate bone metabolism by increasing bone mineral content, density, and osteoblast activity, potentially through the gut–bone axis. These mechanisms suggest that prebiotics may offer a synergistic approach for managing osteoporosis by simultaneously supporting gut health and regulating bone homeostasis.

## 3. Probiotics and Osteoporosis

Probiotics are live microorganisms that confer health benefits to the host by promoting a balanced gut microbiota and improving its characteristics [67]. Osteoporosis has been linked to changes in gut microbiota, particularly a reduction in *Lactobacillus* species and an increase in pro-inflammatory cytokines such as TNF-α, IL-6, and IL-14 in the serum [68]. The concept of bone microbiology highlights the close relationship between gut health and bone metabolism [69]; moreover, as research progresses, scientists are investigating how probiotic exposure affects bone properties and the underlying mechanisms involved (Table 2, Figure 2).

Among the probiotics studied, *Lactobacillus* species have been extensively researched for their role in regulating postmenopausal osteoporosis through the gut–bone axis. Guo et al. administered *Lactobacillus rhamnosus GG* (*LGG*) to ovariectomized rats and observed significant changes in bone formation and gut microbiota [22]. LGG treatment reduced bone loss, improved trabecular microstructure, and increased gut microbiota diversity compared to controls. It also lowered the *Firmicutes*/*Bacteroidetes* ratio, improved estrogen-deficiency-induced inflammation, and enhanced the expression of tight junction proteins in the gut. Additionally, LGG treatment helped balance Th17 and Treg cells in bone tissue, counteracting the osteoporosis caused by estrogen deficiency.

Similarly, Li et al. showed that *Lactobacillus plantarum* treatment in SD rats increased bone density, trabecular number, and thickness while inhibiting osteoclast formation and promoting osteoblast activity [70]. These effects were accompanied by increased gut microbiota diversity and a reduced *Firmicutes*/*Bacteroidetes* ratio, as observed in previous studies. Other research has demonstrated that *Lactobacillus* treatments can prevent bone loss, accelerate fracture healing, and reduce bone density loss in various osteoporosis models [77].

In human studies, elderly women who supplemented with *Lactobacillus reuteri ATCC PTA 6475* experienced reduced bone loss, particularly those with low bone density. Gut microbiota diversity and inflammatory markers also improved [74]. In addition to *Lactobacillus*, other probiotics such as *Bacillus subtilis*, *Bifidobacteria*, and *Prevotella* have been shown to improve gut symptoms and alleviate osteoporosis. These probiotics help reduce gut inflammation and prevent bone loss.

For example, Lan et al. administered *Bifidobacterium lactis BL-99* to mice with ulcerative colitis, showing that BL-99 not only reduced inflammatory cytokines (TNF-α, IL-1β, IL-6, IL-17) but also improved bone volume, trabecular number, and thickness compared to controls [71]. Zhang et al. observed similar results, with *Bifidobacterium* treatment improving bone density, reducing bone loss, and positively altering gut microbiota composition [72]. In human studies, *Bifidobacterium lactis Probio-M8* improved bone metabolism in postmenopausal women by increasing vitamin D3 levels and reducing parathyroid hormone and procalcitonin levels [75].

Moreover, Zhang et al. demonstrated that *Prevotella histicola* could prevent ovariectomy-induced bone loss by promoting osteoblast formation and suppressing osteoclastogenesis in mice while improving gut microbiota composition and diversity [73]. Similarly, *Bacillus subtilis C-3102*, as shown by Takimoto et al., prevented bone loss in postmenopausal women by improving bone density and regulating gut microbiota composition [76].

In addition, probiotic metabolites also play a crucial role in the effects on bone health. Tryptophan metabolites (e.g., indole-3-propionic acid) and polyamines (e.g., spermidine) modulate osteoclastogenesis via aryl hydrocarbon receptor (AhR) signaling and mitochondrial biogenesis, respectively [78,79]. Conversely, trimethylamine N-oxide (TMAO) exacerbates bone resorption by activating the ROS-dependent NF-kappaB signaling pathway [80].

A summary and analysis of estrogen deficiency, glucocorticoid-induced, and ulcerative colitis osteoporosis models revealed that probiotic supplementation reduced the *Firmicutes*/*Bacteroidetes* ratio in ovariectomized mice and glucocorticoid-induced osteoporosis mouse models. Additionally, in the ovariectomized mouse model, probiotics also increased the relative abundance of *Lactobacillus*, *Clostridium*, and *Bifidobacterium* in the gut, while also decreasing the relative abundance of *Desulfovibrio* and *Ruminococcus*. This modulation improved estrogen deficiency-induced inflammatory responses, enhanced the expression of intestinal tight junction proteins, and influenced bone metabolism. In the glucocorticoid-induced osteoporosis mouse model, probiotic supplementation led to a reduction in the relative abundance of *Desulfovibrionaceae* in the gut microbiota, which, in turn, altered bone metabolism. In the ulcerative colitis mouse model, probiotic treatment resulted in a decrease in the relative abundance of *Bacteroides* and *Firmicutes* while increasing the relative abundance of *Akkermansia*. These changes ultimately led to alterations in bone volume percentage, trabecular number, and thickness in the mice.

In summary, probiotics, particularly *Lactobacillus* and *Bifidobacterium* species, play a critical role in osteoporosis management by modulating gut microbiota composition, reducing systemic inflammation, and influencing bone metabolism. Probiotics restore microbial diversity, promote the growth of beneficial gut microbes, enhance gut barrier function, and modulate immune responses. In animal models, *Lactobacillus rhamnosus GG* and *Lactobacillus plantarum* have demonstrated the ability to reduce bone loss, improve trabecular microstructure, and stimulate osteoblast activity while suppressing osteoclastogenesis. These effects are largely mediated through the reduction of pro-inflammatory cytokines like TNF-α and IL-6, which are elevated in osteoporosis and contribute to bone resorption. Additionally, probiotics can influence the gut–bone axis by modulating the *Firmicutes–Bacteroidetes* ratio, which is linked to improved bone health. The interaction between probiotics and immune cells, particularly Treg and Th17 cells, supports bone protection by regulating bone remodeling processes. Probiotics may also mitigate estrogen deficiency-induced inflammation, thus preventing bone loss in postmenopausal models. Overall, probiotics have a multifaceted impact on bone health through their ability to restore gut microbiota balance, reduce inflammation, and directly modulate bone metabolism, positioning them as promising candidates for osteoporosis treatment.

However, a limited number of randomized controlled trials have suggested that the results regarding the improvement of bone mineral density (BMD) markers with probiotic supplementation may be contradictory [81]. For instance, postmenopausal women who consumed *Lactobacillus*-rich probiotics exhibited conflicting results concerning bone density and bone turnover biomarkers [82]. Compared to the control group, the experimental group showed a reduction in bone density loss in the lumbar spine, femoral neck, and trochanter. However, no significant differences were observed in bone turnover biomarkers. One possible reason for this could be the influence of individual differences on the effectiveness of probiotic treatments. It was suggested that probiotics may only have a positive effect on bone health in populations with lower BMD, older age, or higher body mass index (BMI) [83,84]. For example, Gregori et al. recruited 292 early postmenopausal women and administered *Limosilactobacillus reuteri 6475* [83]. After two years, they measured the relative changes in tibial total volumetric BMD, as well as lumbar spine and total hip BMD. The results indicated a significant decrease in BMD for all participants, with no notable differences observed between the groups. However, a re-treatment study involving patients with high BMI found significant therapeutic effects on bone health. This discrepancy may be explained by the fact that the participants in the initial study were early postmenopausal women, whose BMD may not have experienced significant decline at the time of the study. In contrast, individuals with higher BMI may have been more responsive to probiotic treatment due to differing baseline conditions or bone metabolism rates.

## 4. Natural Active Substances and Osteoporosis

Several naturally active substances have shown protective effects on bone health, potentially through modulation of the gut microbiota (Table 3, Figure 2). Kang et al. treated mice with Korean Red Ginseng (KRG) extract and found that it significantly prevented antibiotic-induced bone loss, reduced gut microbiota alpha diversity, and improved gut permeability [85]. They observed shifts in the relative abundance of specific gut bacteria, including *Lactobacillus* and *Alistipes finegoldii*, which may contribute to the gut–bone axis effects of KRG. Further studies confirmed that KRG could also prevent glucocorticoid-induced bone loss in mice by restoring gut microbiota composition [86].

Other natural substances, such as *Agastache rugosa* ethanol extract (EEAR), compound deer bone extract (CBDE), and grape seed extract (GSE), have also demonstrated potential in preventing osteoporosis. Hong et al. showed that EEAR treatment improved bone strength and reversed gut microbiota dysbiosis in ovariectomized mice, suggesting its potential for treating postmenopausal osteoporosis [87]. GSE treatment in estrogen-deficient mice inhibited bone marrow adipose tissue expansion, promoted bone formation, and modulated gut microbiota by reducing pathogenic bacteria and increasing beneficial bacteria such as *Bifidobacteria* [25]. Similarly, CBDE treatment improved trabecular microstructure and gut microbiota composition in ovariectomized mice [88].

While many studies focus on how natural substances, particularly plant-derived compounds, impact bone health through gut microbiota modulation, it is also essential to recognize that these substances may exert direct effects through molecular interactions with the body. Phytoestrogens, for example, mimic estrogen and can bind to estrogen receptors, potentially influencing bone metabolism by modulating bone resorption and formation, especially in postmenopausal women [90]. Additionally, natural compounds such as flavonoids, polyphenols, and alkaloids may act directly on bone cells or hormonal pathways to promote bone health, independent of microbiota-related mechanisms [91]. These compounds interact with various signaling pathways involved in bone remodeling, including those related to osteoblast differentiation and osteoclast activity.

In human studies, a clinical trial supplementing postmenopausal women with hops extract standardized in 8-Prenylnaringenin (HE), along with calcium and vitamin D3, demonstrated that HE supplementation increased bone density and altered gut microbiota composition, with specific bacteria showing positive correlations with bone density [89]. These findings suggest that HE extract may help prevent bone density loss in postmenopausal women, although longer-term studies are needed to confirm these results.

In conclusion, prebiotics, probiotics, and natural active substances show promise in preventing and treating osteoporosis through their effects on gut microbiota and bone metabolism. These findings highlight the need for further research into their clinical applications and potential for enhancing bone health.

## 5. Perspective

Modulating the gut microbiota has emerged as a promising approach for preventing and treating various diseases, including osteoporosis. This review highlights how microbiota modulation can alleviate osteoporosis and offers insights into potential therapeutic strategies. However, our current understanding remains limited.

While existing research emphasizes the critical role of the gut microbiota in osteoporosis, the precise mechanisms by which it influences bone metabolism are still unclear. Future studies should focus on how gut microbes affect bone health through their metabolites, signaling pathways, and immune modulation. Delving deeper into the molecular mechanisms of the gut–bone axis is essential for developing more targeted interventions, which will pave the way for new research directions and clinical applications in osteoporosis prevention and treatment.

Given the significant variations in gut microbiota composition among individuals, personalized intervention strategies are crucial. Future research should explore how personalized treatments, based on an individual’s unique microbiota profile, can optimize therapeutic outcomes. Variations in microbiota composition and immune system function significantly influence the effectiveness of microbiota-based treatments for osteoporosis. The gut microbiota is highly personalized, shaped by factors such as genetics, diet, lifestyle, and pre-existing health conditions [92]. Additionally, immune system variations—such as differences in cytokine profiles and inflammatory responses—affect the body’s response to microbiota modulation [93]. These factors underscore the need for strategies that consider both microbiota composition and immune system status to optimize osteoporosis treatment outcomes.

Much of the current research has focused on short-term effects, while the long-term impacts and safety of microbiota modulation have not been systematically evaluated. Therefore, future studies should investigate the long-term effects and potential side effects of these interventions to ensure their sustained efficacy and safety. A large portion of current research on the gut–bone axis and microbiota-based interventions relies on animal models, particularly rodents. While these models provide controlled environments to study mechanisms and therapeutic effects, they often fail to fully replicate the complexity of human microbiota and bone physiology. Differences in gut microbiota composition between species, along with variations in immune responses and bone metabolism, may limit the generalizability of findings from animal studies to human conditions. Additionally, methods of microbiota manipulation (e.g., antibiotics or fecal microbiota transplantation) used in animals may not be directly applicable to human clinical settings. Therefore, well-designed human clinical trials are crucial for assessing the long-term safety, efficacy, and potential side effects of microbiota-based interventions.

While most studies have concentrated on the immediate effects of microbiota modulation, the long-term consequences remain underexplored. Individuals with compromised immune systems, such as those with HIV or undergoing immunosuppressive treatments, may be at higher risk of opportunistic infections when using probiotics or undergoing fecal microbiota transplantation (FMT). Additionally, although FMT shows promise as a treatment, it carries the risk of transferring harmful pathogens from donor to recipient, underscoring the need for strict safety protocols and careful donor selection.

## 6. Conclusions

The gut microbiota plays a crucial role in the onset and progression of osteoporosis. Current evidence suggests that prebiotics, probiotics, and natural active substances have significant potential to mitigate osteoporosis. However, while early findings are promising, future research must focus on understanding the underlying mechanisms of these interventions and their long-term efficacy. By doing so, we can establish a stronger scientific foundation for microbiota-based osteoporosis treatments. Further exploration will enable the development of more precise and effective intervention strategies, offering new perspectives and methods for preventing and treating osteoporosis.

## 7. Lay Summary

Osteoporosis is a condition where bones become weak and fragile, leading to a higher risk of fractures. Recent studies have found that the balance of microbes in our gut may play a key role in bone health. These tiny organisms affect how our immune system works, how nutrients are absorbed, and how inflammation is controlled. This study looks at how certain dietary changes, like adding prebiotics (which feed good bacteria in the gut), probiotics (live helpful bacteria), and natural substances from plants, might help improve bone strength by supporting a healthy gut. These findings could offer new ways to protect bone health, especially for groups at higher risk, like older adults and women after menopause.

## Figures and Tables

**Table 1:** Summary of studies related to the effects of prebiotics on osteoporosis.

Class	Study Population	Prebiotics	Function	Gut Microbiota	Reference
Animal Experiment	High-fat diet male mice (C57BL/6)	FOS, GOS	FOS/GOS treatment significantly attenuates high-fat-induced bone loss and reverses the imbalance of osteoblast, adipocyte, and osteoclast differentiation. FOS/GOS treatment significantly improves high-fat diet-induced downregulation of intestinal connexin (tight junction protein 1, tight junction protein 15, ZO-1, and JAM-A) expression and an increase in inflammatory factors (TNF-α, IL-6, IL-17).	FOS/GOS treatment significantly ameliorates high-fat diet-induced ecological imbalance of the gut microbiota: increased *Firmicutes*–*Bacteriodetes* and decreased biodiversity.	[24]
	Forty-five male senescence-accelerated mice	5% FOS, 5% GM	Femoral calcium content was significantly higher in the FOS group than in the control group.	The number of *Lactobacillus* and *Bacteroides* anomalies in the cecum contents of mice in the FOS group was significantly higher than that in the control group. *Clostridium* spp. counts were significantly higher in the GM group of mice than in the control group.	[59]
	Female rats (SD)	Short-chain fructo-oligosaccharides	Short-chain fructo-oligosaccharides treatment leads to significantly higher bone anabolic responses with no effect on catabolic parameters and enhanced peak bone mass		[23]
	4-week-old male rats (SD)	GOS	GOS supplementation increases net calcium absorption in a dose-dependent manner. GOS dietary supplementation increases net magnesium absorption, calcium absorption in the femur, calcium and magnesium retention, and fracture strength of the femur and tibia. Increased total bone mineral density and area of the distal femur and bone mineral density of the proximal tibia after dietary supplementation with GOS.	Increased relative ratio of *Bifidobacteria* to GOS. Significant reduction in the number of gut flora bands in mice after GOS dietary supplementation.	[60]
	Female mice (ddY)	Resistant starch (RS)	Upregulation of IL-10 mRNA expression in the colon in mice with 12% resistant starch in the diet. Downregulation of nuclear factor kB receptor activator ligand and IL-7 receptor gene expression in bone marrow of mice with 12% resistant starch in the diet. Dietary 12% resistant starch attenuates ovariectomy-induced bone loss.	Significant increase in the number of *Bifidobacteria* in the feces of mice after dietary supplementation with resistant starch.	[61]
	8-week-old female mice (ddY)	Soya isoflavones (ISO), resistant starch (RS)	Combined treatment with ISO and RS alters the expression of ovariectomy-induced inflammation-associated factors in bone marrow and prevents the loss of trabecular bone density in the distal femur. The combined treatment of ISO and RS can weaken bone resorption in mice.	Resistant starch treatment significantly increases the relative abundance of *Bifidobacterium* spp.	[62]
	12-month-old senile female mice (C57BL/6)	D-mannose	Significant increase in cortical bone volume and bone trabecular microarchitecture in mice following dietary addition of D-mannose. Downregulation of cytokines related to osteoclastogenesis in the bone marrow of mice in the D-mannose group	D-mannose reconstitutes the gut microbiota and alters metabolite composition in mice. Higher proportions of *Verrucomicrobia*, *Akkermansia*, and *Verrucomicrobiaceae* in the feces of D-mannose-supplemented mice compared to Sham group mice. D-mannose increases the relative abundance of *Verrucomicrobia* and *Akkermansiaceae* in feces compared to control mice.	[63]
	10-week-old female rats (SD)	Puerarin	Increased bone mineral density in orally administered Puerarin rats.	Oral intake of Puerarin improves intestinal mucosal integrity and reduces systemic inflammation in rats. Oral intake of Puerarin ameliorates intestinal dysbiosis and increases the concentration of metabolites and short-chain fatty acids in rats; enrichment of amino acid metabolism, LPS biosynthesis, and butyric acid metabolism pathways.	[64]
	28-day-old male mice (ICR)	Xylo-oligosaccharides (XOS)	Significantly higher bone mineral density and fracture strength in mice with 4% XOS in the diet. BMD was significantly enhanced in mice with 4% XOS in the diet during late growth. Dietary supplementation with XOS upregulates the expression of related calcium transport proteins.	XOS significantly decreased cecum pH and increased cecum wall weight in a dose-dependent manner. As XOS concentration increases, villus height and the ratio of villus height to crypt depth increase.	[65]
Population Studies	Healthy adolescent girls	GOS	Both low-dose (5 g GOS/d) and high-dose (10 g GOS/d) GOS treatments significantly improved calcium absorption compared with the control group.	Total dominant fecal flora is not affected by GOS. *Bifidobacteria* in feces increased with treatment with GOS.	[66]

**Table 2:** Summary of studies related to the effects of probiotics on osteoporosis.

Class	Study Population	Probiotics	Function	Gut Microbiota	Reference
Animal Experiment	3-month-old female rats (SD)	*Lactobacillus rhamnosus* GG (*LGG*)	Protective effects of *LGG* were found in ovariectomized rats and were more favorable in osteogenesis. *LGG* treatment ameliorated estrogen deficiency-induced inflammation and mucosal injury and increased GLP-2R and tight junction protein expression.	*LGG* treatment increased the abundance and community diversity of gut microbiota and improved community richness. *LGG* treatment in rats decreased the relative abundance of *Firmicutes* and *Desulfobacterota* and increased the relative abundance of *Bacteroidetes* and decreased the relative abundance ratio of *Firmicutes*/*Bacteroidetes*.	[22]
	12-week-old female rats (SD)	*Lactobacillus plantarum*	*Lactobacillus plantarum* treatment restored bone microstructural parameters, increased bone density, number, and thickness of bone trabeculae. *Lactobacillus plantarum* inhibits osteoclast formation and promotes osteoblast formation.	*Lactobacillus plantarum* treatment increased the diversity of gut microbiota, decreased the ratio of *Firmicutes*/*Bacteroidota*, increased the relative abundance of beneficial bacteria, and decreased the relative abundance of harmful bacteria (*Desulfovibrionaceae*). The serum metabolites of rats in different treatment groups were significantly changed, mainly in the pentose and glucuronic acid interconversion pathway and the propionic acid metabolism pathway.	[70]
	6-week-old male mice (C57BL/6)	*Bifidobacterium lactis* BL-99	*Bifidobacterium lactis* BL-99 treatment significantly improved bone volume percentage (BV/TV), trabecular number, and thickness in mice with ulcerative colitis.	*Bifidobacterium lactis* BL-99 treatment significantly increased the expression of intestinal barrier-related proteins. After *Bifidobacterium lactis* BL-99 treatment, the relative abundance of *Bacteroides* and *Firmicutes* at the family level decreased. The relative abundance of *Akkermansia* increased at the genus level.	[71]
	8-week-old female mice (C57BL/6)	*Bifidobacterium*	*Bifidobacterium* treatment significantly improved bone mineral density, bone volume/total volume ratio (BV/TV), and trabecular number, and effectively inhibited bone loss. *Bifidobacteria* can inhibit the expression of inflammatory cytokines in the intestinal tract, reduce intestinal inflammation, and inhibit the overproduction of osteoclasts.	*Bifidobacterium* treatment increased the relative abundance of *Lactobacillus*, *Clostridium*, and *Bifidobacterium* and decreased the relative abundance of *Desulfovibrio* and *Ruminococcus* in the colon.	[72]
	8-week-old female mice (C57BL/6)	*Prevotella histicola* (*Ph*)	*Ph* treatment inhibited osteoclast formation and promoted osteogenesis, reduced the release of pro-inflammatory cytokines (IL-1β and TNF-α), and reversed the expression of tight junction proteins.	*Ph* treatment improves the composition, richness, and diversity of the gut microbiota. *Ph* treatment can repair intestinal mucosal barrier damage and optimize intestinal permeability.	[73]
Population Studies	Olderwomen	*Lactobacillus reuteri* ATCC PTA 6475	*Lactobacillus reuteri* ATCC PTA 6475 treatment can reduce bone loss in elderly women with low BMD.	The gene richness of the gut microbiota was significantly higher after *Lactobacillus reuteri* ATCC PTA 6475 treatment.	[74]
	Patients with postmenopausal osteoporosis	*Bifidobacterium lactis* Probio-M8	*Bifidobacterium lactis* Probio-M8 treatment can improve bone metabolism, as indicated by increased levels of vitamin D3 and decreased levels of parathyroid hormone and procalcitonin in serum.	*Bifidobacterium lactis* Probio-M8 treatment affected the interact-related network of gut microbiota, especially bacteria that produce short-chain fatty acids. *Bifidobacterium lactis* Probio-M8 treatment significantly increased genes encoding carbohydrate metabolic pathways and genes encoding choline phosphocytidylate transferase.	[75]
	Healthy postmenopausal women	*Bacillus subtilis* C-3102 (C-3102)	C-3102 significantly increased BMD in the total hip. The bone resorption marker tartrate-resistant acid phosphatase isoform 5b tended to decrease after 12 weeks of C-3102 treatment.	After 12 weeks of C-3102 treatment, the relative abundance of the *Bifidobacterium* genus significantly increased. The relative abundance of the *Fusobacterium* genus was significantly reduced at 12 and 24 weeks of C-3102 treatment.	[76]

**Table 3:** Summary of studies related to the effects of naturally occurring substances on osteoporosis.

Class	Study Population	Natural-Occurring Substance	Function	Gut Microbiota	Reference
Animal Experiment	12-week-old male mice (Balb/C)	Korean Red Ginseng extract (KRG)	KRG can prevent antibiotic-induced bone loss.	KRG can prevent the reduction of α diversity of gut microbiota and the increase of intestinal permeability in mice. Several genera, including *Lactobacillus*, *rc4-4*, and *Alistipesfinegoldii*, may be involved in the effect of KRG on the axis of the gut.	[85]
	7-week-old male mice (CD-1)	Korean Red Ginseng extract (KRG)	Treatment with KRG extract prevented trabecular bone loss in the distal femur.	It causes significant changes in gut microbiota.	[86]
	7-week-old female mice (C57BL/6)	*Agastache rugosa* ethanol extract (AR)	AR treatment suppressed bone strength loss. AR treatment elevated osteogenic markers in bone marrow cells and type 1 collagen α1 in the distal femur.	AR treatment reversed the disturbance of gut microbiota induced by ovariectomy.	[87]
	11-week-old female mice (C57BL/6)	Grape seed extract (GSE)	GSE treatment can inhibit the expansion of bone marrow adipose tissue, restore lipolysis of bone marrow adipose tissue, and promote bone formation, thereby improving bone loss.	GSE treatment could reduce the number of opportunistic pathogens (*Alistipes*, *Turicibacter*, and *Romboutsia*), increase the number of beneficial bacteria *Bifidobacterium*, and regulate the imbalance of gut microbiota caused by ovariectomy. GSE mainly affects lipid and amino acid metabolism.	[25]
	Female mice (ICR)	Compound deer bone extract	CBDE treatment could significantly improve the microstructure of the trabecular bone in mice. The trabecular bone increased, and the reticular structure was clearer and more orderly.	CBDE treatment makes the gut microbiota of osteoporosis model mice tend towards healthy mice in terms of type and quantity.	[88]
Population Studies	PostmenopausalWomen	Hop Extract Standardized in 8-Prenylnaringenin (HE)	Forty-eight weeks of HE supplementation increased whole-body bone mineral density.	In the HE group, a higher abundance of Turicibacter and Shigella genera (which are thought to be associated with whole-body bone mineral density) was observed.	[89]

## Data Availability

No new data were created or analyzed in this study.
